# Unraveling the role of long non-coding RNAs in chronic heat stress-induced muscle injury in broilers

**DOI:** 10.1186/s40104-024-01093-6

**Published:** 2024-10-08

**Authors:** Zhen Liu, Yingsen Liu, Tong Xing, Jiaolong Li, Lin Zhang, Liang Zhao, Yun Jiang, Feng Gao

**Affiliations:** 1 College of Animal Science and Technology, Key Laboratory of Animal Origin Food Production and Safety Guarantee of Jiangsu Province, Jiangsu Collaborative Innovation Center of Meat Production and Processing, Quality and Safety Control, Nanjing Agricultural University, Nanjing, 210095 People’s Republic of China; 2 Anhui Key Laboratory of Livestock and Poultry Product Safety Engineering, Institute of Animal Husbandry and Veterinary Medicine, Anhui Academy of Agricultural Sciences, Hefei, 230031 China; 3 Institute of Agro-Products Processing, Jiangsu Academy of Agricultural Sciences, Nanjing, 210014 People’s Republic of China; 4 School of Food Science and Pharmaceutical Engineering, Nanjing Normal University, Nanjing, 210023 People’s Republic of China

**Keywords:** Apoptosis, Broiler, Chronic heat stress, LncRNA, Muscle

## Abstract

**Background:**

Chronic heat stress (CHS) is a detrimental environmental stressor with a negative impact on the meat quality of broilers. However, the underlying mechanisms are not fully understood. This study investigates the effects of CHS on long non-coding RNA (lncRNA) expression and muscle injury in broilers, with a focus on its implications for meat quality.

**Results:**

The results showed that CHS diminished breast muscle yield, elevated abdominal fat deposition, induced cellular apoptosis (*P* < 0.05), and caused myofibrosis. Transcriptomic analysis revealed 151 differentially expressed (DE) lncRNAs when comparing the normal control (NC) and HS groups, 214 DE lncRNAs when comparing the HS and PF groups, and 79 DE lncRNAs when comparing the NC and pair-fed (PF) groups. After eliminating the confounding effect of feed intake, 68 lncRNAs were identified, primarily associated with cellular growth and death, signal transduction, and metabolic regulation. Notably, the apoptosis-related pathway P53, lysosomes, and the fibrosis-related gene *TGF-β2* were significantly upregulated by lncRNAs.

**Conclusions:**

These findings indicate that chronic heat stress induces cellular apoptosis and muscle injury through lncRNA, leading to connective tissue accumulation, which likely contributes to reduced breast muscle yield and meat quality in broilers.

## Introduction

Since the nineteenth century, humans have produced a significant amount of greenhouse gases, leading to a continuous rise in Earth’s temperature and frequent occurrences of extreme heat events [1, 2]. All homeothermic animals have a thermoneutral zone. When the temperature exceeds this zone, the animal cannot maintain a steady body temperature and heat stress occurs. Heat stress can be categorized into two categories: acute heat stress (AHS), which is brief (lasting for a few hours to days) and sudden exposure to extremely high ambient temperature, causing physiological and metabolic changes that intend to maintain its survival; and chronic heat stress (CHS), which is caused by prolonged (lasting for several days to weeks) exposure to high ambient temperature, and the animal will develop a certain level of adaptability to the environment [3]. These days, one of the biggest challenges facing animal husbandry is heat stress.

The poultry industry plays a significant role in global food production, with broilers being one of the primary sources of meat protein. Continuous directional breeding, propelled by market forces, has markedly enhanced the growth rate of broilers. According to the National Chicken Council, in 1925, the market weight of a broiler was 1.13 kg (market age 112 d). By 2023, this weight had increased to 2.97 kg (market age 47 d) [4]. The faster growth rate is accompanied by increased metabolic heat production. Moreover, because broilers primarily dissipate heat through respiration, they cannot efficiently regulate their body temperature, rendering them susceptible to high temperatures. As a result, prolonged high temperatures in summer frequently cause chronic heat stress in broilers. Research indicates that chronic heat stress reduces the growth and slaughter performance of chicken, as well as decreases meat quality [5, 6]. Our previous study found that chronic heat stress (32 °C, 14 d) induces vacuolar degeneration, cellular apoptosis, muscle damage, decreases the springiness, and increases the hardness of broilers [7]. However, the mechanism of abnormal meat induced by chronic heat stress remains incompletely comprehended.

In recent years, studies have identified that heat stress can induce cell apoptosis and organ dysfunction or injury. Exposure to 32 °C for 21 d induced myodegenerative changes, perivenular CD3^+^ cell infiltration, and lipidosis, exacerbating the severity of white striping in broiler chickens [8]. Under chronic heat stress conditions (34 °C for 12 h/d over 21 d), White Recessive Rock chickens exhibited inflammatory cellular hyperplasia and localized inflammatory lesions in the leg muscles [9]. Chronic heat stress (34 °C for 8 h/d over 21 d) led to Gln deficiency, oxidative damage, and decreased meat quality in the thigh muscles of broilers [10].

Long non-coding RNAs (lncRNAs) have garnered considerable attention as critical regulatory factors within cells. Representing a subclass of non-coding RNAs with lengths exceeding 200 nucleotides, constituting approximately 80% of non-coding RNAs [11]. Within cells, lncRNAs exert regulatory effects through interactions with DNA, mRNA, miRNA, and proteins [12]. Developing evidence suggests that lncRNAs play pivotal roles in stress response, metabolism, apoptosis, proliferation, and cell cycle progression [13, 14]. Research in recent years has identified the effects of heat stress on broiler meat quality from the perspectives of physiological, metabolic, cellular, and molecular responses, but few reports have focused on the lncRNA differences in pectoralis muscle under heat stress.

In this study, we examined the impact of CHS on the pectoralis major (PM) muscle of broilers, employing Illumina HiSeq6000 sequencing to investigate alterations in lncRNA expression in the breast muscle under CHS conditions. Our findings will enhance our understanding of the mechanisms by which CHS impacts the pectoralis major muscle in broilers.

## Materials and methods

### Experimental design, animals, and management

All broiler testing procedures had been approved by the Animal Care and Use Committee of Nanjing Agricultural University (approved protocol number: SYXK 2021-0086). As previously published [7], three experimental groups were established, each consisting of 48 chickens distributed into 6 replicates. Notably, there were no discernible differences in the body weight among the three groups. The temperature of the chambers for the normal control (NC) and pair-fed (PF) groups was set at 22 °C, while the temperature for the heat stress (HS) group was set at 32 °C. Birds in the NC and HS groups had ad libitum access to feed, whereas the PF group received an amount of feed equal to the intake of the HS group from the previous day. Humidity was maintained at 55% to 60%. The experiment lasted for 14 d.

### Respiration rate and rectal temperature

At 10:00 on d 1, 3, 7, and 14 of the experiment, respiratory rate was determined by visually measuring the number of breaths per minute in a calm state. Two broilers per replicate were randomly chosen, and their respiratory rate was calculated as the average of measurements taken simultaneously by 2 observers. Subsequently, an electronic thermometer was employed to measure their rectal temperature.

### Sample collection

At 42 days old, 12 broilers, 2 birds per replicate, were selected from each group. Subsequently, the selected birds were humanely slaughtered following electrical stunning. The broilers were then plucked and segmented, and carcass characteristics were evaluated following the approach outlined by Zhu et al. [15]. Samples of the PM muscle were taken and kept in liquid nitrogen for subsequent analyses. The samples were obtained along the longitudinal orientation of the muscle fibers and rapidly frozen in liquid nitrogen for frozen slicing. Additionally, muscle samples of identical dimensions were collected and fixed in 4% paraformaldehyde solution for paraffin slicing.

### Sirius Red staining

The muscle samples underwent dehydration using gradient ethanol, followed by treatment with paraformaldehyde, and subsequent embedding in paraffin prior to being sectioned into 6 µm slices. Then, it was stained according to the methods in the literature [16]. Briefly, the sections were stained using Sirius Red staining solution and rapidly dehydrated with anhydrous ethanol. Subsequently, the sections were immersed in clean xylene for 5 min for transparency and sealed with neutral gum. Image acquisition was performed with a BX50 microscope (Olympus, Tokyo, Japan).

### Assay for caspase-3 activity

A commercial caspase-3 activity kit (G015-1-3, Nanjing Jiancheng Bioengineering Institute, China) was used to assess the activity of caspase-3 in the PM muscle of broiler. Briefly, 50 mg of PM muscle was homogenized in 50 μL lysis buffer, and then centrifugation at 6,000 × *g* for 10 min. Subsequently, the supernatants were analyzed. Protein concentration was determined using a BCA kit (C503061-1250, Sangon Biotech, Shanghai, China).

### TUNEL assay

The muscle samples were embedded in optimum cutting temperature compound and frozen on the quick-freezing table of the slicer. Subsequently, sections of 8 µm thickness were obtained and mounted onto glass slides. The sections were then stained using a TUNEL detection kit (A112-03, VazumE, Nanjing, China) following the manufacturer’s instructions. Fluorescence microscopy was immediately employed to capture images. The ratio of positively stained (green) nuclei was calculated to ascertain the proportion of apoptotic cells.

### LncRNA extraction, sequencing, and analysis

The total RNA from PM muscle was extracted using a TRIzol reagent (Invitrogen, Carlsbad, CA, USA). Then, rRNAs were removed using an Illumina Ribo-Zero Gold kit (MRZG12324, Illumina, San Diego, CA, USA) to retain mRNAs and ncRNAs. The enriched mRNAs and ncRNAs were reverse transcribed into complementary DNA (cDNA) to construct a cDNA library, which was then subjected to sequencing using the Illumina NovaSeq 6000 platform.

Fastp was used to filter the reads produced by the NovaSeq 6000 platform in order to produce clean, high-quality reads. Subsequently, the reads mapped to the ribosomal RNA (rRNA) database were eliminated by using the Bowtie2. The clean reads were aligned to the reference genome, followed by transcript reconstruction for the identification of new genes and splice variants. Potential lncRNAs were identified using the CNCI [17] and CPC [18] software. Principal component analysis (PCA) was performed to verify the accuracy of the sequencing data and operational stability. FPKM was used to conduct the initial measurement of lncRNA expression levels. This step was primarily for data normalization and preliminary exploratory analysis. Then, raw count data was used as input for differential expression (DE) analysis with DESeq2. LncRNAs with a significance level of *P* < 0.05 and an absolute fold change ≥ 1.5 were deemed as differentially expressed.

Subsequently, through association analysis with our previously published miRNA and mRNA datasets [19], miRNA targets were predicted using three software tools: mireap, miRanda, and TargetScan. The ceRNA network was constructed by evaluating mRNA-miRNA and lncRNA-miRNA correlations using the Spearman Rank correlation coefficient (SCC) with SCC < –0.7, and lncRNA-mRNA correlations using the Pearson correlation coefficient (PCC) with PCC > 0.9. Significant common miRNA sponges were identified using a hypergeometric test (*P* < 0.05).

The lncRNA-miRNA-mRNA network was then assembled from the identified co-expression triplets and visualized using Cytoscape. Finally, all target mRNAs identified in the ceRNA interaction network underwent KEGG pathway enrichment analysis and GO analysis.

### Quantitative real-time PCR analysis

A commercial reverse transcription kit (R323-01, Vazyme Biotech Co., Ltd., Nanjing, China) was employed to convert RNA into cDNA. Primer5 software was used to design primer pairs (Table 1). Quantitative real-time PCR was carried out on a PCR instrument (QuantStudio5, Applied Biosystems, Foster City, CA, USA) using a SYBR qPCR kit (Q711-02, Vazyme Biotech Co., Ltd., Nanjing, China). The 2^−ΔΔCt^ method was used to calculate lncRNA expression levels, with β-actin as the reference gene.

**Table 1:** Primer sequences

Gene ID	Primer (5´→3´)
MSTRG.12376.1	F: AGAACCGACCGTAAGCCACTTG
	R: AACCTGCCAAATCCCTCCCAATC
MSTRG.13528.7	F: AAGCAAGCCAAGCAAGCCAAC
	R: CAGTGAGTCCAGCGAGCAGTTTC
MSTRG.9586.1	F: AGCGACCTCCACTTCCAACC
	R: GCACACAGCACAGCCTTAGTC
MSTRG.1229.2	F: CAATCTGTGCTCTGCTCCTTAGTAG
	R: CACTTGCCACCCTGACAACTC
MSTRG.5339.3	F: TGTTGGTGAAGATGTAGTGCTGAAG
	R: CCGTCCTGTGTGCTCCTCTC
β-actin	F: ACGTCGCACTGGATTTCGAG
	R: TGTCAGCAATGCCAGGGTAC

### Statistical analysis

The results, presented as mean ± SD, underwent one-way ANOVA analysis in SPSS Statistics 20.0 (SPSS Inc., Chicago, IL, USA). Duncan multiple range test was utilized to assess differences, with statistical significance set at *P* < 0.05. The data were analyzed with the bird as the statistical unit (*n* = 12).

## Results

### Chronic heat stress's effects on respiration rate and rectal temperature

**Fig. 1:**
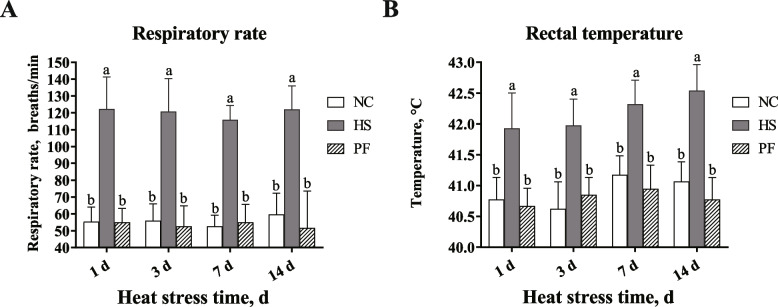
Effects of chronic heat stress on respiratory rate and rectal temperature of broilers. **A** Effects of chronic heat stress on respiratory rate of broilers. **B** Effects of chronic heat stress on rectal temperature of broilers. NC, The normal control group. HS, The heat stress group. PF, The pair-fed group. The result represents as mean value ± standard deviation (SD) (*n* = 12). ^a,b^Significant differences are represented by different letters (*P* < 0.05)

The respiratory rate and rectal temperature of broilers in 3 experimental groups are shown in Fig. 1. Throughout the study period, the respiratory rate of broilers in the NC and PF groups remained consistently within the range of 40 to 70 breaths/min. Conversely, broilers in the HS group exhibited markedly higher respiratory rate, ranging between 110 and 140 breaths/min, which was statistically significant difference compared to the NC and PF groups (*P* < 0.05). Similarly, the rectal temperature of broilers in the NC and PF groups consistently remained between 40 and 41 °C throughout the experiment, while the broilers subjected to heat stress experienced rectal temperatures exceeding 42 °C, which was a significant elevation compared to the NC and PF groups (*P* < 0.05).

### Chronic heat stress decreased carcass characteristics

The carcass characteristics of broilers in the three groups are presented in Table 2. The body weight of the NC group was significantly higher than that of both the HS group and the PF group (*P* < 0.05), with no significant difference observed between the HS and PF groups (*P* > 0.05). Prolonged exposure to a high temperature of 32 °C led to a notable reduction in breast muscle yield and a substantial increase in abdominal fat deposition (*P* < 0.05). Chronic heat stress had no discernible impact on the dressed yield, half-eviscerated yield, all-eviscerated yield, and thigh muscle yield (*P* > 0.05).

**Table 2:** Effects of chronic heat stress on carcass characteristics of broilers

Items	Group			*P*-value
	NC	HS	PF	
Body weight, g	2,637.5 ± 201.32^a^	2,391.67 ± 233.16^b^	2,455.83 ± 184.23^b^	0.02
Dressed yield, %	95.23 ± 0.94	95.28 ± 0.66	95.10 ± 0.58	0.82
Half-eviscerated yield, %	90.73 ± 1.36	91.26 ± 0.75	91.44 ± 0.76	0.21
All-eviscerated yield, %	78.46 ± 2.21	78.86 ± 1.49	79.32 ± 1.33	0.47
Breast muscle yield, %	26.61 ± 1.45^a^	24.87 ± 1.27^b^	26.25 ± 2.01^a^	0.03
Thigh muscle yield, %	18.10 ± 0.81	18.61 ± 0.44	17.84 ± 1.35	0.14
Abdominal fat yield, %	1.61 ± 0.28^b^	2.13 ± 0.29^a^	1.32 ± 0.42^c^	< 0.01

*NC* The normal control group, *HS* The heat stress group, *PF* The pair-fed group. The result represents as mean value ± standard deviation (SD) (*n* = 12). ^a,b^Significant differences are represented by different letters (*P* < 0.05)

### Chronic heat stress induces muscle cell apoptosis in broilers

**Fig. 2:**
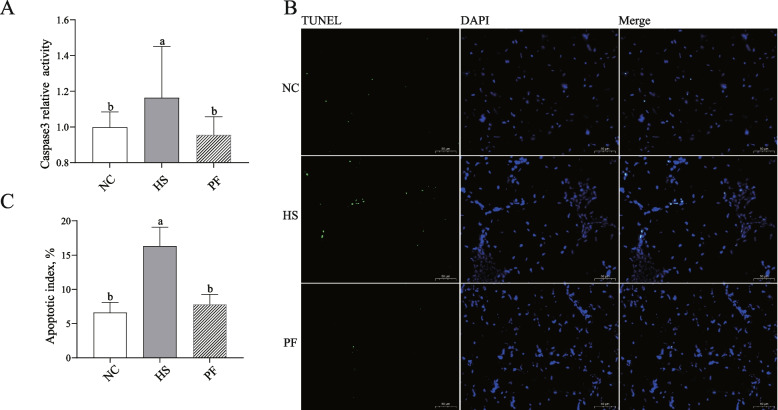
Effects of chronic heat stress on muscle cell apoptosis in broilers. **A** The caspase3 activity of the pectoralis major (PM) muscle in the three groups. **B** and **C** The TUNEL staining of the PM muscle in the three groups. ^a,b^Different letters represent significant differences (*P*< 0.05). NC, The normal control group. HS, The heat stress group. PF, The pair-fed group. The result represents as mean value ± standard deviation (SD) (*n* = 12). ^a,b^Significant differences are represented by different letters (*P* < 0.05)

The caspase-3 activity and TUNEL staining of the PM muscle are shown in Fig. 2. Chronic heat stress markedly elevated caspase-3 activity in the PM muscle of broilers (*P* < 0.05) (Fig. 2A). Additionally, chronic heat stress led to a substantial increase in the proportion of TUNEL-positive cells in the PM muscle, as illustrated in Fig. 2B and C (*P* < 0.05).

### Chronic heat stress induces damage in the pectoralis major muscle

**Fig. 3:**
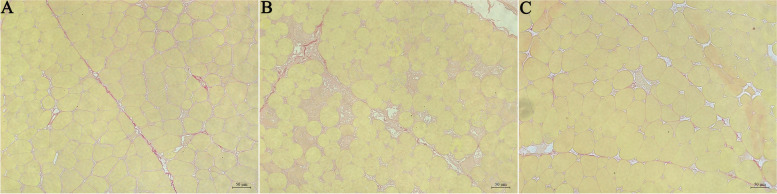
The Sirius Red staining of the pectoralis major (PM) muscle. **A**–**C** The Sirius Red staining of the PM muscle in the NC, HS and PF groups. Bar = 50 μm. NC, The normal control group. HS, The heat stress group. PF, The pair-fed group

The Sirius Red staining results of chicken breast muscle are depicted in Fig. 3. The findings suggest that following 14 days of heat stress, the HS group displayed more dispersed muscle fibers compared to the other 2 groups. Moreover, some cells exhibited vacuolation and even ruptured, while collagen fibers occupied extensive areas, indicating the occurrence of muscle damage.

### Heat stress causes notable alterations in lncRNAs expression patterns

**Fig. 4:**
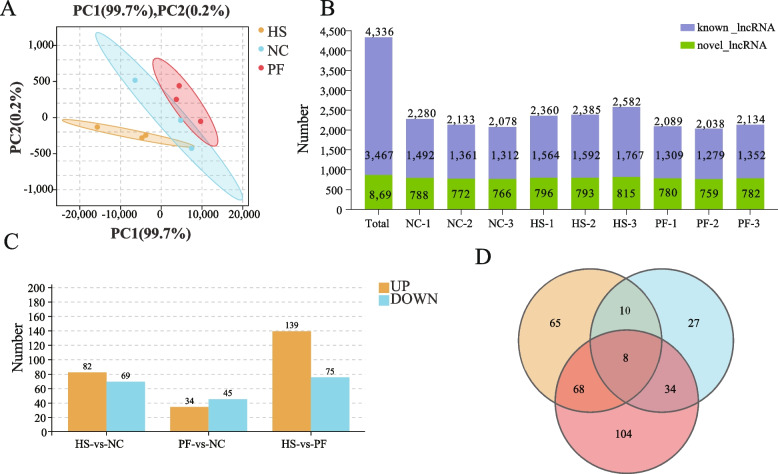
Effects of chronic heat stress on lncRNA expression profile in the pectoralis major muscle of broilers. **A** Principal component analysis (PCA) of the samples. **B** The lncRNA species identification in the pectoralis major (PM) muscle of broilers. **C** Identification of differentially expressed lncRNA in the PM muscle of broilers in three groups. **D** Venn analysis of differentially expressed lncRNA. NC, The normal control group. HS, The heat stress group. PF, The pair-fed group

In this study, PCA was used to analyze the lncRNA expression datasets. The PCA results (Fig. 4A) showed that the first principal accounted for 99.7% of the variability in the RNA-Seq dataset. In total, 4,336 lncRNAs were identified across three groups, encompassing 3,467 known lncRNAs and 869 novel lncRNAs (Fig. 4B). Comparative analysis revealed 82 elevated and 69 reduced DE lncRNAs in the HS group relative to the NC group. Similarly, relative to the PF group, 139 elevated and 75 reduced DE lncRNAs were identified in the HS group. Furthermore, in contrast to the NC group, the PF group exhibited 45 reduced and 34 elevated DE lncRNAs (Fig. 4C). Our previous studies had established that chronic heat stress can affect broilers in two ways [5]. To understand the effects of the non-feed intake factor under chronic heat stress on the breast muscle of broilers, and further explore the impact of non-feed intake factor on the PM muscle, 68 DE lncRNAs were screened out through the Venn analysis (Fig. 4D). Among them, 47 lncRNAs were elevated and 21 lncRNAs were reduced in the HS group in comparison to the other two groups.

### GO and KEGG pathway enrichment analyses of DE lncRNAs

LncRNA can function as ceRNA, sequestering miRNAs and thereby modulating gene translation. Utilizing our previous research data [19], we established the regulatory network of lncRNA-miRNA-mRNA to enhance our comprehension of the roles played by the 68 DE lncRNAs triggered by non-feed intake factors during chronic heat stress. The analysis found that 167 up-regulated mRNAs and 98 down-regulated mRNAs were regulated by lncRNA.

**Fig. 5:**
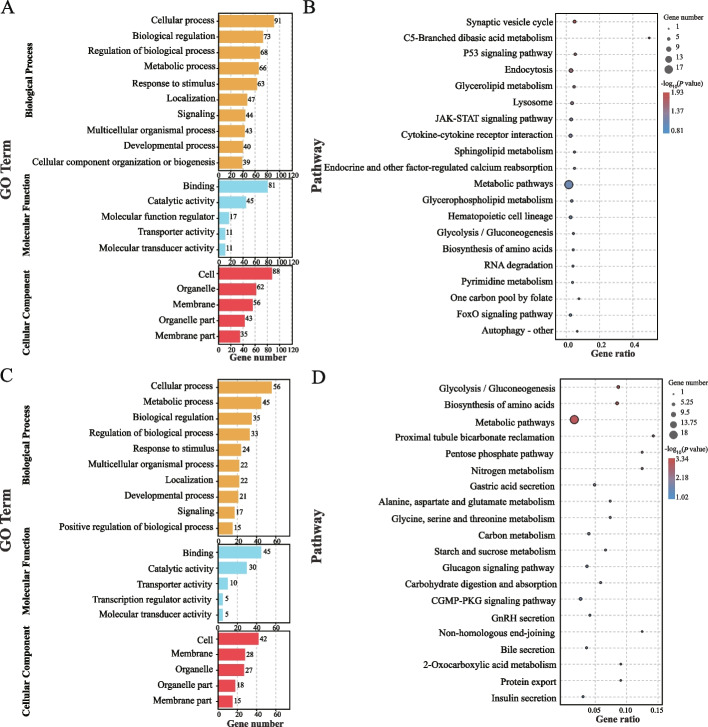
GO and KEGG pathway enrichment analysis of DE lncRNA. **A** GO function analysis of up-regulated lncRNA. **B** Pathway enrichment analysis of up-regulated lncRNA. **C** GO function analysis of down-regulated lncRNA. **D** Pathway enrichment analysis of down-regulated lncRNA

Following the identification of DE lncRNAs, a comprehensive analysis was conducted to elucidate their functional implications. The GO analysis revealed that for the up-regulated lncRNA, the top three enriched terms in the biological process (BP) category were cellular process, biological regulation, and regulation of biological process. Similarly, in the molecular functions (MF) category, predominant enrichment was observed in binding and catalytic activity. Concurrently, the cellular component (CC) category highlighted enrichments in cell, organelle, and membrane components (Fig. 5A), delineating the intricate involvement of these lncRNAs in fundamental cellular activities. Furthermore, the KEGG pathway analysis provided insights into the potential pathways influenced by these DE LncRNAs. The analysis demonstrated the top 20 enriched pathways, predominantly associated with pivotal cellular processes such as cell growth and death, signal transduction, and metabolic regulation. Notable pathways included the synaptic vesicle cycle, C5-Branched dibasic acid metabolism, P53 signaling pathway, endocytosis, glycerolipid metabolism, lysosome, JAK-STAT signaling pathway, cytokine-cytokine receptor interaction, sphingolipid metabolism, endocrine and other factor-regulated calcium reabsorption, metabolic pathways, glycerophospholipid metabolism, hematopoietic cell lineage, glycolysis/gluconeogenesis, biosynthesis of amino acids, RNA degradation, pyrimidine metabolism, one carbon pool by folate, FoxO signaling pathway, and autophagy - other (Fig. 5B).

For the down-regulated lncRNAs, the GO analysis revealed that in the BP category, the top three enriched terms were cellular process, metabolic process, and biological regulation. Within the MF category, binding and catalytic activity emerged as the predominant enrichment GO terms. Cell, membrane, and organelle were the top three enriched terms in the CC category (Fig. 5C). Furthermore, KEGG pathway analysis indicated that the top 20 enriched pathways were predominantly involved in metabolic regulation. These pathways include glycolysis/gluconeogenesis, biosynthesis of amino acids, metabolic pathways, pentose phosphate pathway, nitrogen metabolism, alanine, aspartate and glutamate metabolism, starch and sucrose metabolism, glycine, serine and threonine metabolism, glucagon signaling pathway, carbon metabolism, carbohydrate digestion and absorption, 2-oxocarboxylic acid metabolism, and insulin secretion (Fig. 5D).

**Fig. 6:**
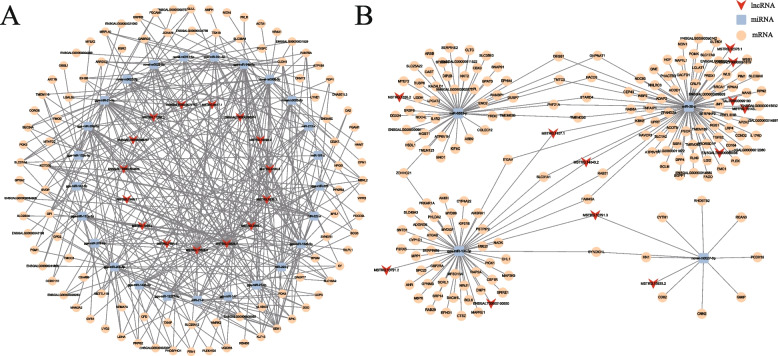
The ceRNA network of differentially expressed lncRNAs. **A** The ceRNA network of down-regulated lncRNA. **B** The ceRNA network of up-regulated lncRNA

Next, we visualized the ceRNA regulatory network, by analyzing the expression profiles and interactions of lncRNAs, miRNAs, and mRNAs, we constructed ceRNA networks which included 136 lncRNA-miRNA relationship pairs and 447 miRNA-mRNA relationship pairs (Fig. 6). Through this construction and subsequent analysis, we identified miR-26a and gga-miR-10c-5p as central nodes within the network. Several lncRNAs may exert significant regulatory functions through their interactions with these miRNAs. For instance, lncRNAs MSTRG.1407.1 and MSTRG.14543.2 interact with miR-26a, influencing the expression of *TGFβ2*, *FADD*, and *CCND2*, while also affecting *MYD88*, *SERPINB5*, and *CTSZ* expression through interactions with gga-miR-10c-5p. These genes are all implicated in apoptosis pathways. Additionally, we identified several lncRNAs associated with oxidative stress and metabolic processes. For example, lncRNA MSTRG.13528.7 interacts with gga-miR-21-5p and gga-miR-20a-5p, regulating the expression of *UCP3* and *UQCRB*, respectively. lncRNA MSTRG.7561.1 modulates *IDH3B* expression through interaction with gga-miR-34a-5p, while MSTRG.5339.2 regulates *GYS1* expression via gga-miR-212-5p.

### qRT-PCR validation of the lncRNA

**Fig. 7:**
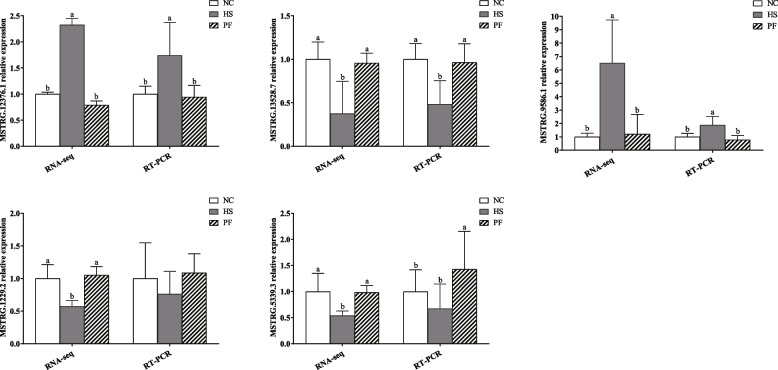
The qRT-PCR validation of the differentially expressed (DE) lncRNAs. The data that are shown as the mean ± standard deviation (SD) values. NC, Normal control group; HS, The heat stress group; PF, The pair-fed group. ^a,b^Different letters represent significant differences (*P *< 0.05)

Five lncRNAs were randomly selected for validation using qRT-PCR. The observed relative expression levels of these five lncRNAs were in line with the trends observed in the RNA sequencing data (Fig. 7). These findings provided further validation and reinforced the reliability of our transcriptomic dataset.

## Discussion

Over the past decades, the average temperature of the Earth’s surface has steadily risen, making heat stress a significant environmental stressor with detrimental effects on animal welfare and livestock production. Due to the absence of sweat glands, birds increase their respiratory rate to dissipate heat as ambient temperatures rise [20]. When the house temperature surpasses the critical threshold, broilers lose thermal homeostasis, causing their body temperature to elevate [21]. Therefore, respiratory rate and rectal temperature were utilized to assess whether broilers were experiencing heat stress [22]. Throughout the experimental period, broilers subjected to heat stress exhibited significantly elevated respiratory rates and rectal temperatures than those observed in the NC and PF groups. These findings strongly indicated that the broilers were indeed experiencing heat stress.

Research across various species has demonstrated that heat stress can induce apoptosis in muscle cells, leading to muscular damage. Chronic heat stress induces endoplasmic reticulum stress-mediated apoptosis by activating the PERK-calcineurin-MuRF1 signaling pathway, promoting protein catabolism in broilers, thereby impairing their growth performance [23]. Heat stress (34 ± 1 °C for 8 h and 22 ± 1 °C for the remaining day) induces mitochondrial and muscle damage through the PINK1-Parkin signaling pathway, thereby reducing muscle quality [24]. Liu et al. [7] reported that chronic heat stress induced cell apoptosis and muscle injury in the PM muscle of broilers. Consistent with these findings, our experiment revealed a significant increase in caspase3 activity and TUNEL-positive cells following fourteen days of heat stress, indicating the induction of cell apoptosis in the PM muscle of broilers. Under normal circumstances, muscle damage undergoes a repair process, including stages such as muscle fiber regeneration, proliferation of scar tissue (connective tissue), maturation of regenerated muscle fibers, scar tissue contraction, and reorganization. Following these stages, muscle function is restored. In the vast majority of cases, skeletal muscle injuries heal without forming functionally disabling fibrotic scars. However, severe muscle trauma or repeated injuries can lead to fibrotic scarring, resulting in muscle fibrosis [25, 26]. To further evaluate the extent of muscle injury, we employed Sirius Red staining. Our findings unveiled that apoptosis triggered by chronic heat stress perpetuated substantial harm to the breast muscles, fostering the development of scar tissue. This persistent damage likely elucidates the decline in breast muscle yield, the escalation in shear force values and hardness, and the diminished springiness attributed to chronic heat stress [7].

Despite extensive research on the effects of heat stress on the PM muscle of broilers, the specific mechanism governing apoptosis and muscle injury induced by chronic heat stress remains unclear. LncRNA serves as a ceRNA, regulating gene expression by interacting with miRNA and playing crucial roles in various biological processes and stress responses [27]. In this research, transcriptome was employed to investigate the changes of lncRNA expression in the PM muscle. Previous research has indicated that chronic heat stress can affect broilers through feed intake factor and non-feed intake factor (high temperature) [5]. We established the PF group to eliminate the influence of feed intake and investigate the effects of non-feed intake factor under chronic heat stress. The PCA result indicated that chronic heat stress reprogrammed lncRNA expression in broiler PM muscle, with non-feed intake factor (high temperature) emerging as dominant determinant. Following the exclusion of feed intake factor, we identified 68 DE lncRNAs attributed to non-feed intake factors. In order to unravel the role of these DE lncRNAs, we constructed a lncRNA-miRNA-mRNA ceRNA regulatory network. encompassing 136 lncRNA-miRNA and 447 miRNA-mRNA relationship pairs. Through the network analysis, we identified miR-26a as central nodes within the network. Research indicates that the miR-26 family plays a crucial role in apoptosis [28]. Therefore, lncRNAs targeting miR-26a may significantly contribute to the regulation of apoptosis. This network construction provides a holistic view of the complex regulatory interactions among lncRNAs, miRNAs, and mRNAs, shedding light on potential regulatory pathways and molecular mechanisms underlying the observed phenotypic changes in response to chronic heat stress.

Subsequently, we performed KEGG pathway enrichment analysis and found that apoptosis-related pathways were significantly enriched, particularly the P53 and lysosome pathways. The P53 pathway has been shown to mediate cellular stress responses [29]. A variety of stress signals, such as DNA damage, oncogene expression, and hypoxia, can activate the P53 pathway, which in turn regulates multiple processes, including energy metabolism, genomic stability, antioxidant functions, and apoptosis [30]. Salinas et al. [31] demonstrated that stresses could induce germ cell apoptosis through the P53 pathway. Exposure to copper (II) and/or arsenite can induce immunosuppression and apoptosis via the NF-κB/p53 signaling pathway [32].Li et al. [33] discovered that heat stress-induced ROS increased P53 phosphorylation at Ser46, resulting in the activation of the mitochondrial apoptosis pathway. PM_2.5_ exposure upregulated the phosphorylation level of myocardial P53, resulting in an increased *Bax*/*Bcl-2* expression, which subsequently elevated caspase-3 levels, leading to cardiomyocyte apoptosis and myocardial injury [34]. Lysosome, single-membrane sac-like organelles containing acid hydrolases, are essential for decomposing cellular material and maintaining cellular homeostasis [35]. Membrane permeabilization of lysosomes can be triggered by stress, further significantly contributes cell death, including apoptosis, pyroptosis, and ferroptosis [36]. When the lysosomal membrane is disrupted, the lysosomal proteases are released into the cytosol, activating caspases and inducing apoptosis [37, 38]. Liu et al. [39] found that Bisphenol A treatment blocked autophagy flux through disrupting the normal function of lysosome and induced apoptosis. Yi et al. [40] found that heat stress increased intracellular ROS, caused lysosomal membrane permeabilization, released cathepsin B to the cytosol, and induced apoptosis of epithelial cells in the small intestine. *TGF-β* is a cytokine with various biological roles, including the cellular differentiation, apoptosis, immunological function, wound repair, and tissue fibrosis [41, 42]. Existing studies have shown that *TGF-β2* activation can lead to fibrosis in the lungs and liver [43, 44]. In this study, chronic heat stress activates the P53 and lysosome pathways. Furthermore, we identified several lncRNAs acting as ceRNA, activating *TGF-β2* by sequestering miR-26-Z. Additionally, increased caspase-3 activity and apoptosis were observed. Sirius Red staining indicated damage to PM muscle resulting from chronic heat stress, accompanied by an elevation in collagen fibers, this was similar to Patael’s findings [45]. These findings suggest that chronic heat stress may activate the P53 pathway and lysosomal signaling pathway through lncRNA, inducing apoptosis of PM muscle cells, resulting in sustained damage to the PM muscles and activation of *TGF-β2*, thereby triggering PM muscle fibrosis. This could be one of the reasons for the decrease in PM muscle yield and the increase in hardness.

## Conclusion

In summary, chronic heat stress induced cell apoptosis, muscle injury, and altered lncRNA expression in PM muscle. A total of 68 lncRNAs were selected which were induced by high temperature rather than by feeding intake. Functional analysis revealed that the identified 68 lncRNAs primarily participate in metabolism and apoptosis. Among them, the apoptosis-related pathways P53 and lysosomes, as well as the fibrosis-related gene *TGF-β2* were significantly upregulated. Moreover, we screened out some ceRNA networks might be associated with apoptosis induce by chronic heat stress. Our research deepens the understanding of how chronic heat stress decrease the breast muscle yield and meat quality of broilers, while also providing new insight for environmental risk assessment.

## Data Availability

All data generated or analyzed during this study are available from the corresponding authors on reasonable request. These sequence data have been submitted to the National Center for Biotechnology Information (NCBI, https://www.ncbi.nlm.nih.gov) Sequence Read Archive databases under accession number PRJNA899658.

## Abbreviations

ceRNA: Competing endogenous RNA
CHS: Chronic heat stress
DE lncRNAs: Differentially expressed lncRNAs
GO: Gene Ontology
KEGG: Kyoto Encyclopedia of Genes and Genomes
LncRNA: Long non-coding RNA
NC: Normal control
PF: Pair-fed
PCA: Principal component analysis
PM: Pectoralis major
ROS: Reactive oxygen species
